# Effect of tofogliflozin on obesity-related health problems in patients with type 2 diabetes and overweight or obesity—a post-hoc sub-analysis of the UTOPIA study

**DOI:** 10.1007/s13340-025-00845-7

**Published:** 2025-08-18

**Authors:** Naoto Katakami, Tomoya Mita, Takafumi Masuda, Yasunori Sato, Hirotaka Watada, Iichiro Shimomura

**Affiliations:** 1 Department of Metabolic Medicine, Osaka University Graduate School of Medicine, 2-2, Yamadaoka, Suita, Osaka 565-0871 Japan; 2 Department of Metabolism and Endocrinology, Juntendo University Graduate School of Medicine, Hongo 2-1-1, Bunkyo-ku, Tokyo, 113-8421 Japan; 3 Department of Preventive Medicine and Public Health, Keio University School of Medicine, 45 Shinanomachi, Shinjuku-ku, Tokyo, 160-8582 Japan

**Keywords:** Diabetes, Obesity, Overweight, Sodium-glucose cotransporter 2 inhibitor, Tofogliflozin

## Abstract

**Objective:**

This study aimed to evaluate the effects of tofogliflozin, a sodium-glucose cotransporter 2 inhibitor, on health issues in patients with type 2 diabetes (T2DM) and overweight or obesity in real-world clinical practice.

**Methods:**

This post-hoc sub-analysis of the UTOPIA trial, a randomized prospective study, included 210 patients (102 in the tofogliflozin group and 108 in the conventional treatment group) with a body mass index of ≥ 25.0 kg/m^2^. The primary outcome was the percentage of patients achieving a weight loss of ≥ 3% at 26 and 104 weeks. Secondary outcomes included improvements in obesity-related health problems.

**Results:**

At 26 weeks, 46.4% of the tofogliflozin group (95% confidence interval [CI] 36.2–56.8%) achieved a weight loss of 3% of more, significantly higher than 14.4% in the conventional treatment group (95% CI 8.3–22.7%, p < 0.001). At 104 weeks, 62.0% of the tofogliflozin group (95% CI 51.2–71.9%) achieved this outcome compared with 29.3% in the conventional treatment group (95% CI 20.6–39.3%, p < 0.001). The tofogliflozin group also showed greater improvements in glycated hemoglobin, fasting blood glucose, blood pressure, liver indices, high-density lipoprotein-cholesterol, serum uric acid, and quality of life (QOL). Additionally, arterial stiffness progression was significantly reduced (p < 0.05) and the increase in urinary albumin tended to be attenuated (p = 0.056) in the tofogliflozin group.

**Conclusions:**

In Japanese patients with T2DM and obesity, tofogliflozin effectively promotes weight loss and has a positive impact on various obesity-related health problems and QOL. These findings suggest its potential as a therapeutic option for improving both metabolic and cardiovascular health in this population.

**Trial registration:**

UMIN000017607 (https://www.umin.ac.jp/icdr/index.html).

**Supplementary Information:**

The online version contains supplementary material available at 10.1007/s13340-025-00845-7.

## Introduction

Sodium-glucose cotransporter 2 (SGLT2) inhibitors improve blood glucose levels by selectively inhibiting SGLT2, which plays an important role in renal glucose reabsorption, thereby increasing urinary glucose excretion. SGLT2 inhibitors may to be particularly useful for patients with obesity and type 2 diabetes mellitus (T2DM) as they are expected to reduce visceral adiposity and body weight due to the energy loss associated with glucose excretion. Moreover, they may improve several obesity-related health problems, such as hyperglycemia, dyslipidemia, hypertension, hyperuricemia, atherosclerosis, renal dysfunction, cardiac dysfunction, and hepatic dysfunction [1–9]. However, few studies have directly evaluated the effects of SGLT2 inhibitor treatment on obesity and obesity-related health problems in patients with obesity and T2DM. Particularly regarding tofogliflozin, a SGLT2 inhibitor commonly used in Japan [10], few studies have evaluated the long-term effects of tofogliflozin on body weight and obesity-related health problems in patients with overweight and obesity along with T2DM.

The “Using TOfogliflozin for Possible Better Intervention against Atherosclerosis for type 2 diabetes patients (UTOPIA)” trial, a randomized clinical trial to investigate the preventive effects of tofogliflozin on the progression of atherosclerosis in patients with T2DM, revealed that tofogliflozin did not delay the progression of carotid artery intima-media thickness (IMT); however, it significantly improved arterial stiffness, glycemic control, body weight, body mass index (BMI), abdominal circumference, systolic blood pressure, high-density lipoprotein cholesterol (HDL-C) levels, and patients’ quality of life (QOL) during the 2-year intervention period [11–14].

Moreover, based on the post-hoc sub-analysis of the UTOPIA trial, we recently reported that the beneficial effects of tofogliflozin on circulating levels of hepatic enzymes, uric acid, and hemoglobin (Hb) lasted for 2 years in patients with T2DM [15]. Furthermore, a prospective observational 2-year extension study of the UTOPIA trial also indicated that such beneficial effects on various cardiovascular risk factors persisted for ≥ 4 years [16].

However, the UTOPIA study was not specifically designed to target only individuals with T2DM and overweight or obesity. Therefore, to evaluate the effect of tofogliflozin on various health problems in patients with overweight or obesity and T2DM in real-life clinical practice, we conducted a post-hoc analysis using data extracted exclusively from participants in the UTOPIA study whose baseline BMI was ≥ 25.0 kg/m^2^.

## Materials and methods

### Study design

This post-hoc sub-analysis was based on data obtained from the UTOPIA trial. The study design of the original UTOPIA trial has been described previously [11]. Briefly, the UTOPIA trial was a 2-year prospective, randomized, open-label trial to evaluate the efficacy of tofogliflozin in preventing the progression of atherosclerosis in patients with T2DM. All randomized patients were followed up until the scheduled end of the study, irrespective of adherence to or discontinuation of study medication for any reason. Clinical and biochemical data were collected at 0, 26, 52, 78, and 104 weeks after randomization. The primary outcomes of the original study were changes in the mean IMT of the common carotid artery.

The protocol of the original study was approved by the Ethical Review Board of Osaka University Hospital (approval number: N18007; date of approval: 8/7/2019), and written informed consent was obtained from all patients after a complete explanation of the study. The protocol of this sub-analysis was also approved by the Ethical Review Board of Osaka University Hospital (approval number: 23382; date of approval: 3/5/2024). As this was a post-hoc analysis using only existing materials, it was considered exempt from the requirement for written informed consent from study participants in accordance with the Ethical Guidelines for Medical and Health Research Involving Human Subjects in Japan (partially amended version issued on 2023/3/27). Instead, relevant information regarding the study was open to the public, and opportunities for refusal were ensured. The study was conducted in accordance with the Declaration of Helsinki, Ethical Guidelines for Medical and Health Research Involving Human Subjects, Clinical Trials Act, and other current legal regulations in Japan.

The UTOPIA study was registered with the University Hospital Medical Information Network Clinical Trials Registry, a nonprofit organization in Japan that meets the requirements of the International Committee of Medical Journal Editors (registration number: UMIN000017607).

To avoid bias, data management and monitoring were conducted by a third-party entity (Soiken Inc., Toyonaka, Osaka, Japan).

### Study population

The original inclusion criteria were age between 30 and 74 years, presence of T2DM, and a glycated hemoglobin (HbA1c) level of ≥ 6% but < 9%. The exclusion criteria included presence of type 1 or secondary diabetes mellitus; participant being in the perioperative period; presence of a serious infection or injury; history of myocardial infarction, angina, stroke, or cerebral infarction; estimated glomerular filtration rate (eGFR) of < 30 mL/min/1.73 m^2^ or end-stage renal failure; serious liver function impairment; moderate to severe heart failure; urinary tract or genital infection; pregnancy, possibility of pregnancy, or nursing status; history of hypersensitivity to tofogliflozin; presence or history of a malignant tumor; prohibition from using tofogliflozin; and other reasons for ineligibility determined by an investigator.

Originally, 340 patients who met the eligibility criteria were enrolled at 23 institutions across Japan (Supplementary Material 1) and randomly allocated to either the tofogliflozin group (20 mg of tofogliflozin once daily, n = 169) or the conventional treatment group (those using drugs other than the SGLT2 inhibitor, n = 171). After excluding one patient from further analysis because of lack of data regarding the primary endpoint, 169 and 170 patients in the tofogliflozin and conventional treatment groups, respectively, were included in the full analysis set. Finally, those with a BMI of ≥ 25.0 kg/m^2^ were included in the present analysis.

### Randomization and study intervention

The enrolled participants were randomly assigned to either the tofogliflozin treatment group or the conventional treatment group without SGLT2 inhibitors. In the tofogliflozin group, 20 mg of tofogliflozin once daily was started in addition to ongoing therapy. However, the addition of an alternative antidiabetic agent (excluding another SGLT2 inhibitor) was permitted 12 weeks after randomization. In the conventional treatment group, either the dosage of the ongoing therapy was increased, or a concomitant oral glucose-lowering drug (excluding any SGLT2 inhibitor) was added 12 weeks after randomization. Treatment was continued to achieve the target value specified in the Japanese Treatment Guide for Diabetes [17] (HbA1c < 7.0%) in both groups. In case of hypoglycemia, the dosage of the concomitant oral glucose-lowering drug was titrated.

### Study outcomes

Data changes over time in the tofogliflozin and conventional treatment groups were compared within and between groups. The primary outcome was the percentage of patients in each treatment group achieving a weight loss of least 3% from week 0 to 26 and 104 weeks, and the difference between treatment groups. It was reported that, after 6 months of weight loss guidance for individuals with obesity, the greater the weight loss from pre-intervention, the greater the improvement in blood pressure, blood glucose levels, lipid levels, uric acid levels, and liver function, and that a 3% weight loss from the pre-intervention weight was enough to show significant improvement [18]. Hence, the Japan Society for the Study of Obesity (JASSO) Obesity Treatment Guidelines set the weight loss goal for obesity at 3% of current body weight over a 3–6 month period [19]. Therefore, the primary endpoint of this study was also set at 3% weight loss at 6 months (26 weeks) after the start of the intervention.

Secondary outcomes included achievement rate of HbA1c levels < 7%, systolic blood pressure < 130 mmHg, diastolic blood pressure < 80 mmHg, low-density lipoprotein cholesterol (LDL-C) levels < 120 mg/dL, triglyceride levels < 150 mg/dL, HDL-C levels of ≥ 40 mg/dL, and uric acid levels of 7 mg/dL or less at 0, 26, 52, 78, and 104 weeks in each treatment group; and intra- and inter-group comparisons for changes over time regarding each item (HbA1c, weight, BMI, abdominal circumference, blood pressure, etc.) at 0, 26, 52, 78, and 104 weeks. The incidence of cardiovascular events (ischemic heart disease, cerebrovascular disease, and arteriosclerosis obliterans) and adverse events were also compared between groups.

### Clinical and biochemical assessment

Height, weight, and waist circumference were measured, and BMI was calculated. The determination of obesity (BMI ≥ 25.0 kg/m^2^) was based on the JASSO criteria [16]. Blood pressure was measured at rest with a mercury sphygmomanometer. Blood samples were collected after an overnight fast. HbA1c, glucose, serum lipids (total cholesterol, HDL-C, LDL-C, and triglycerides), creatinine, aspartate transaminase (AST), alanine transaminase (ALT), gamma-glutamyl transpeptidase (γGTP), uric acid, red blood cells (RBCs), Hb, hematocrit (Ht), white blood cells (WBC), and total platelet counts (PLT) were measured using standard techniques. Urinary albumin excretion was measured by the improved bromocresol purple method using a spot urine sample. The eGFR was calculated using the following formula: eGFR (mL/min per 1.73 m^2^) = 194 × age − 0.287 × serum creatinine − 0.1094 (× 0.739 for females).

### Assessment of subclinical atherosclerosis

Carotid IMT, brachial-ankle pulse wave velocity (baPWV), and ankle-brachial index (ABI) were evaluated as indicators of subclinical atherosclerosis.

Ultrasonography scans of the carotid artery were performed by an expert (specifically-trained sonographers) based on the Japan Society of Ultrasonics in Medicine guidelines [20]. Scanning of the extracranial carotid artery was performed bilaterally in different projections, and the site of greatest thickness, including plaque lesions, was sought along the arterial walls. IMT was measured as the distance between two parallel echogenic lines corresponding to the vascular lumen and the adventitial layer. To avoid inter-reader variability, all scans were stored electronically and sent to the IMT evaluation committee. The sent images were inspected in random order by experienced investigators who were not informed of the clinical characteristics of the participants, using automated digital edge detection software (Intimascope; MediaCross, Tokyo, Japan) [21]. The software system averaged approximately 200 points of the IMT values in the segment 2 cm proximal to the dilation of the carotid sinus (mean-IMT-CCA). Additionally, the maximum thicknesses of the intima and media layers, including the plaque lesions, in the common carotid arteries (max-IMT-CCA) were captured separately.

The baPWV and ABI were measured using the same volume plethysmography apparatus (BP-203RPE II form PWV/ABI, Omron Healthcare Co., Ltd., Kyoto, Japan), with patients in the supine position after at least 5 min of rest, as previously reported [22]. Specifically, four oscillometric cuffs, each connected to a plethysmographic sensor that determined the volume of the pulse from and to an oscillometric pressure sensor that measured blood pressure, were wrapped on both the brachia and ankle; two electrocardiogram electrodes were placed on each wrist. The cuffs were simultaneously pressurized to the approximate value of the patient’s diastolic pressure such that the pulse volume waveforms could be recorded using semiconductor pressure sensors. The distance between the sampling points of the baPWV was calculated automatically according to the height of the participant. The path length from the suprasternal notch to the ankle (La) was calculated as follows: La = 0.8129 × height (in cm) + 12.328. The path length from the suprasternal notch to the brachium (Lb) was calculated as follows: Lb = 0.2195 × height − 2.0734. The baPWV was calculated according to the following formula: baPWV = (La − Lb)/Tba, where Tba was the time interval between the wavefront of the brachial waveform and that of the ankle waveform [22]. Two simultaneous measurements of baPWV were recorded on the right and left sides.

### Assessment of diabetes therapy-related QOL

In the current study, we used the Diabetes Therapy-Related Quality Of Life questionnaire (DTR-QOL) 7, a short version of the original DTR-QOL, which comprised seven questions selected from the original 29 items. The total score, after simple addition of the item scores except the Q2 score, was converted to 0–100 (best-case response = 100; worst-case response = 0), with a higher total score after conversion reflecting better treatment satisfaction. The Q2 score, which reflected weight gain with treatment, was separately evaluated, with a higher score indicating better treatment satisfaction. Details are provided in Supplemental Material 2.

### Safety evaluation

All adverse events were recorded during the study and are described in Supplemental Material 3.

### Statistical analysis

All enrolled patients were included in the analysis. Differences between treatment groups regarding the percentage of patients achieving at least 3% weight loss from week 0 to 26 and 104 weeks and in the percentage achieving each treatment goal were evaluated with the χ-squared test, and tests of significance (intra-group comparison) in the percentage achieving goals at each observation point from baseline were evaluated with the McNemar test. Similarly, differences between treatment groups in the percentage of achievement of each secondary outcome (HbA1c levels, blood pressure, serum lipid levels, etc.) at each observation time point were evaluated by the χ-squared test. Moreover, regarding secondary outcomes, the Student’s t test was used to test the null hypothesis that the differences in the measured values and changes in each endpoint in the two groups were equal. The 95% confidence intervals (CIs), based on the t-distribution of the difference between the measured values and the amount of change for each endpoint, were calculated. If the distribution of the measured values deviated significantly from the normal distribution, a t-test or Wilcoxon rank-sum test was performed after log transformation. The significance of change over time (change from baseline) within a treatment group was tested with a one-sample t test. The significance level was set at a two-sided p-level of 0.05, and no multiplicity adjustment was performed. All analyses were performed using SAS software version 9.4 (SAS Institute, Cary, NC, USA).

## Results

**Fig. 1:**
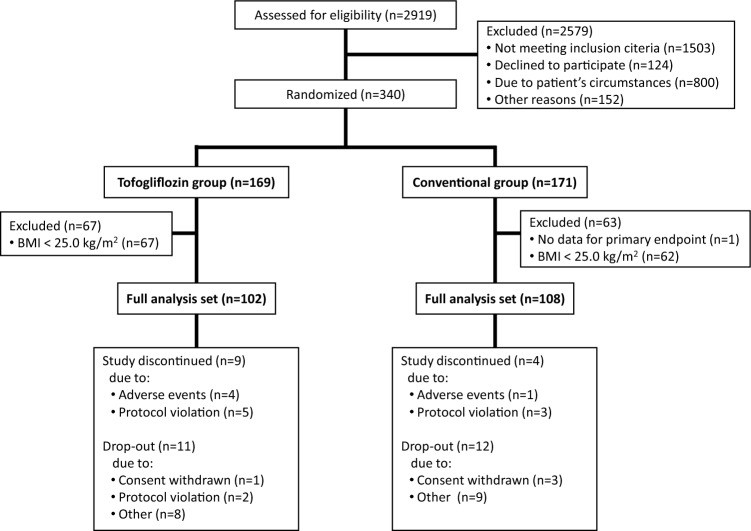
Study flow chart showing patient enrollment and analysis

Among the study patients in the original UTOPIA trial, 210 individuals (102 from the tofogliflozin group and 108 from the conventional treatment group) met the inclusion criterion of the present sub-analysis (BMI ≥ 25.0 kg/m^2^) (Fig. 1). The clinical characteristics of the study population, including concomitant medication use at baseline, are summarized in Table 1. Supplemental Table 1 shows the use of the main concomitant medications during the observation period. Compared with the conventional treatment group, the tofogliflozin group tended to use less dipeptidyl peptidase-4 inhibitors throughout the observation period. However, no significant treatment group differences were observed throughout the observation period with respect to prescription rates of other diabetes medications, including glucagon-like peptide-1 receptor agonists and insulins. Furthermore, compared with the conventional treatment group, the tofogliflozin group tended to use less antihypertensive drugs throughout the observation period. Moreover, there were no differences in the use of lipid-lowering agents between the treatment groups.

**Table 1:** Clinical characteristics of patients in both treatment groups

Parameters	Tofogliflozin group (n = 102)	Conventional group (n = 108)
Sex (males) (%)	63 (61.8)	65 (60.2)
Age (years)	59.3 ± 9.7	58.5 ± 10.3
Current smoking	27 (26.7)	20 (18.5)
Body mass index (kg/m^2^)	30.0 ± 5.4	29.7 ± 3.4
Waist circumference (cm)	99.9 ± 10.8 (n = 90)	99.4 ± 9.8 (n = 98)
Duration of diabetes (years)	11.9 ± 8.5 (n = 95)	11.5 ± 7.6 (n = 103)
HbA1c (%)	7.5 ± 0.8	7.4 ± 0.8
Fasting blood glucose (mg/dL)	144.7 ± 33.0	143.0 ± 34.7 (n = 107)
Hypertension	57 (55.9)	76 (70.4)
Systolic blood pressure (mmHg)	134.0 ± 14.0 (n = 100)	135.5 ± 17.5 (n = 106)
Diastolic blood pressure (mmHg)	78.8 ± 9.5 (n = 100)	80.6 ± 10.7 (n = 106)
Dyslipidemia	69 (67.7)	83 (76.9)
Total cholesterol (mg/dL)	187.5 ± 28.0 (n = 100)	190.1 ± 32.2 (n = 103)
LDL-C (mg/dL)	109.6 ± 25.1	112.8 ± 25.1 (n = 107)
HDL-C (mg/dL)	51.8 ± 12.3	50.8 ± 11.6
Triglyceride (mg/dL)	142.9 ± 82.0	151.7 ± 71.8 (n = 107)
Uric acid (mg/dL)	5.7 ± 1.2 (n = 101)	5.6 ± 1.2 (n = 107)
Diabetic retinopathy	21 (20.8)	17 (15.9)
eGFR (mL/min/1.73 m^2^)	81.0 ± 20.6 (n = 101)	82.4 ± 24.6 (n = 107)
Diabetic nephropathy	37 (36.3)	40 (37.0)
Use of glucose-lowering agents	93 (91.2)	97 (89.8)
Use of antihypertensive drugs	54 (52.9)	67 (62.0)
Use of lipid-lowering agents	55 (53.9)	68 (63.0)
Use of antithrombotic agents	9 (8.8)	11 (10.2)

Data are presented as numbers (%) of patients or means ± standard deviations. Treatment goal achievement rates are expressed as percentage (95% confidence interval)

*HbA1c* glycated hemoglobin, *LDL-C* low-density lipoprotein-cholesterol, *HDL-C* high-density lipoprotein-cholesterol, *eGFR* estimated glomerular filtration rate

### Primary outcome

**Fig. 2:**
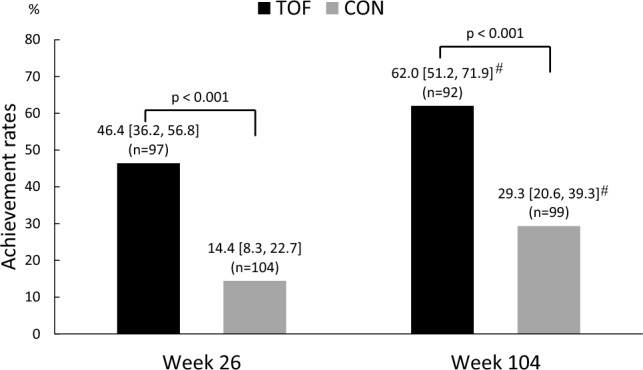
The percentage of patients in each treatment group achieving a weight loss of least 3% from week 0 to 26 and 104 weeks, and the difference between treatment groups. Differences between treatment groups regarding the percentage of patients achieving at least 3% weight loss from week 0 to 26 and 104 weeks and in the percentage achieving each treatment goal were evaluated with the χ-squared test, and tests of significance (intra-group comparison) in the percentage achieving goals at each observation point from baseline were evaluated with the McNemar test. At week 26, the percentage of patients achieving at least 3% weight loss from week 0 was significantly higher in the tofogliflozin group (46.4%; 95% CI 36.2–56.8%) than the conventional treatment group (14.4%; 95% CI 8.3–22.7%) (p < 0.001). At week 104, the percentage of patients achieving at least 3% weight loss from week 0 was also significantly higher in the tofogliflozin group (62.0%; 95% CI 51.2–71.9%) than the conventional treatment group (29.3%; 95% CI 20.6–39.3%) (p < 0.001). Achievement of at least 3% weight loss was significantly higher at week 104 than at week 26 in both the tofogliflozin and conventional treatment groups (tofogliflozin group: p = 0.006; conventional treatment group: p = 0.005). *TOF* tofogliflozin group, *CON* conventional treatment group. ^#^p < 0.01 (McNemar test)

At week 26, the percentage of patients achieving at least 3% weight loss from week 0 was significantly higher in the tofogliflozin group (46.4%; 95% CI 36.2–56.8%) than in the conventional treatment group (14.4%; 95% CI 8.3–22.7%) (p < 0.001) (Fig. 2). At week 104, the percentage of patients achieving at least 3% weight loss from week 0 was also significantly higher in the tofogliflozin group (62.0%; 95% CI 51.2–71.9%) than in the conventional treatment group (29.3%; 95% CI 20.6–39.3%) (p < 0.001) (Fig. 2). Achievement of at least 3% weight loss was significantly higher at week 104 than at week 26 in both the tofogliflozin and conventional treatment groups (tofogliflozin group: p = 0.006; conventional treatment group: p = 0.005).

### Secondary outcomes

**Fig. 3:**
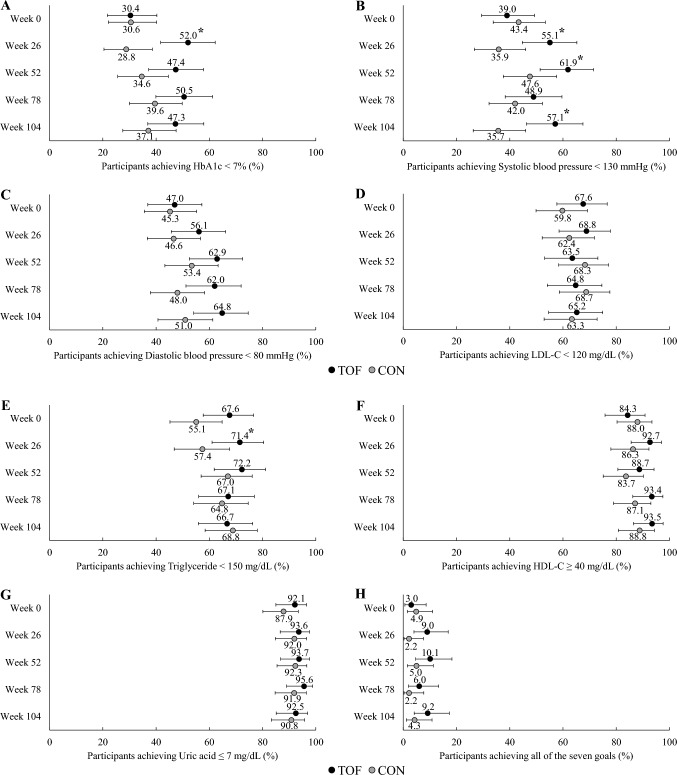
Percentage of treatment goal achievement in both treatment groups at each observation point and the difference between treatment groups. Dots represent achievement rates of each treatment goal as follows; **A** HbA1c < 7%, **B** systolic blood pressure < 130 mmHg, **C** diastolic blood pressure < 80 mmHg, **D** LDL-C < 120 mg/dL, **E** triglyceride < 150 mg/dL, **F** HDL-C ≥ 40 mg/dL, **G** uric acid ≤ 7 mg/dL, and **H** all seven goals from **A** to **G**. Error bars are 95% confidence intervals. *p < 0.05 between the tofogliflozin and conventional treatment groups. *TOF* tofogliflozin group, *CON* conventional treatment group, *HbA1c* glycated hemoglobin, *LDL-C* low-density lipoprotein-cholesterol, *HDL-C* high-density lipoprotein-cholesterol

The achievement rates of each secondary endpoint (HbA1c < 7%, systolic blood pressure < 130 mmHg, diastolic blood pressure < 80 mmHg, LDL-C < 120 mg/dL, triglyceride < 150 mg/dL, HDL-C ≥ 40 mg/dL, and uric acid level ≤ 7 mg/dL) in each treatment group at 0, 26, 52, 78, and 104 weeks, and the differences between treatment groups are shown in Fig. 3 and Supplemental Table 2. The achievement rates of HbA1c (< 7%), systolic blood pressure (< 130 mmHg), and triglyceride (< 150 mg/dL) at week 26 were significantly higher in the tofogliflozin group than in the conventional treatment group. The achievement rates of all secondary endpoints also tended to be higher in the tofogliflozin group than in the conventional treatment group. At week 104, the achievement rate of systolic blood pressure < 130 mmHg was significantly higher in the tofogliflozin group than in the conventional treatment group; however, there were no significant group differences in the achievement rate of the other secondary outcomes.

**Fig. 4:**
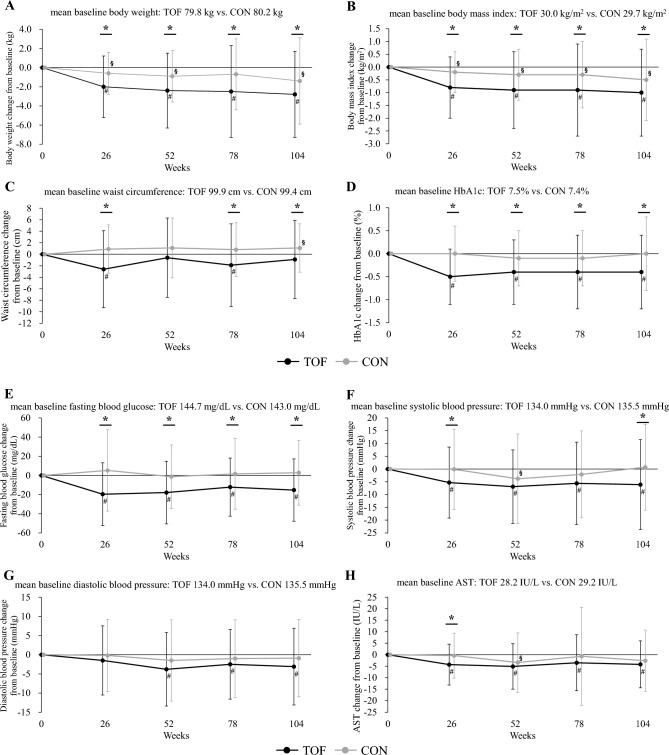

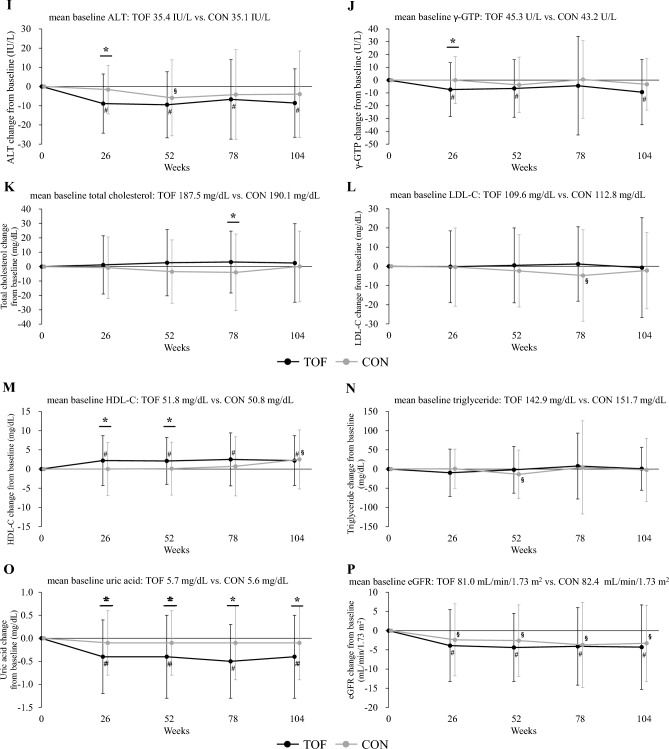

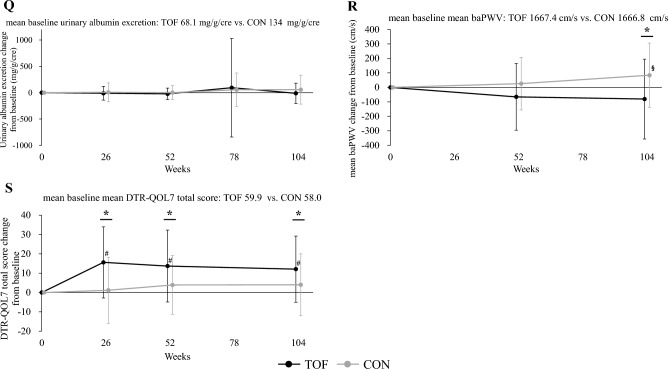
Changes over time and within- and between-group comparisons of clinical parameters at each observation point. **A** Body weight, **B** body mass index, **C** waist circumference, **D** HbA1c, **E** fasting blood glucose, **F** systolic blood pressure, **G** diastolic blood pressure, **H** AST, **I** ALT, **J** γ-GTP, **K** total cholesterol, **L** LDL-C, **M** HDL-C, **N** triglyceride, **O** uric acid, **P** eGFR, **Q** urinary albumin excretion, **R** baPWV, and **S** DTR-QOL7 total score. Data are presented as means, and error bars are standard deviations. *p < 0.05 between the tofogliflozin and conventional treatment groups. ^#^p < 0.05 from baseline values in the tofogliflozin group. ^§^p < 0.05 from baseline values in the conventional treatment group. *TOF* tofogliflozin group, *CON* conventional treatment group, *HbA1c* glycated hemoglobin, *AST* aspartate aminotransferase, *ALT* alanine transaminase, *γ-GTP* gamma glutamyl transferase, *LDL-C* low-density lipoprotein-cholesterol, *HDL-C* high-density lipoprotein-cholesterol, *eGFR* estimated glomerular filtration rate, *baPWV* brachial-ankle pulse wave velocity, *DTR-QOL* diabetes therapy-related quality of life

As shown in Fig. 4 and Supplemental Table 3, in the tofogliflozin group, body weight, BMI, waist circumference, HbA1c, fasting blood glucose, systolic blood pressure, AST, ALT, γGTP, uric acid, and eGFR were significantly decreased, and HDL-C, WBC count, RBC count, Hb, Ht, DTR-QOL7 Q2 score, and DTR-QOL7 total score were significantly increased after the 26-week study period. Regarding the other parameters, no significant changes were observed before or after the 26-week study period. In contrast, in the conventional treatment group, body weight, BMI, and eGFR decreased significantly; however, regarding the other parameters, no significant changes were observed before or after the 26-week study period. As for body weight, BMI, waist circumference, HbA1c, fasting blood glucose, systolic blood pressure, AST, ALT, γGTP, HDL-C, uric acid, RBC count, Hb, Ht, DTR-QOL7 Q2 score, and DTR-QOL7 total score, there was a significant difference between the tofogliflozin group and the conventional treatment group in the amount of change during the 26-week study period; however, regarding the other parameters, there were no significant differences in change between the two groups over the 26-week study period.

As shown in Fig. 4 and Supplemental Table 3, similar results were observed at week 104. In the tofogliflozin group, HbA1c, fasting blood glucose, body weight, BMI, systolic blood pressure, diastolic blood pressure, AST, ALT, γGTP, eGFR, uric acid, mean-IMT-CCA and max-IMT-CCA were significantly decreased, and HDL-C, RBC count, Hb, Ht, adiponectin, and DTR-QOL7 total score were significantly increased after the 104-week study period. Regarding the other parameters, no significant changes were observed before or after the 104-week study period. In contrast, in the conventional treatment group, body weight, BMI, eGFR, mean-IMT-CCA, and max-IMT-CCA decreased significantly, while abdominal circumference, HDL-C, left baPWV, and mean baPWV increased significantly. Regarding the other parameters, no significant changes were observed before or after the 104-week study period. Regarding body weight, BMI, waist circumference, HbA1c, fasting blood glucose, systolic blood pressure, uric acid, RBC count, Hb, Ht, right baPWV, left baPWV, mean baPWV and DTR-QOL7 total score, there were significant differences between the tofogliflozin and conventional treatment groups in the amount of change during the 104-week study period; however, regarding the other parameters, there was no significant difference in change between the two groups over the 104-week study period.

### Safety evaluation

There were no significant differences in the incidence of ischemic heart disease, cerebrovascular disease, arteriosclerosis obliterans, or total mortality between the two groups. There were also no specific adverse events in both groups that could be attributed to the drugs. Moreover, there were no remarkable differences in the incidence of adverse events between the two groups (Table 2).

**Table 2:** Summary of serious adverse events and adverse events

Parameters	Tofogliflozin group (n = 102)	Conventional group (n = 108)
Any adverse events	42 (41.2)	56 (51.9)
Severe adverse events	16 (15.7)	20 (18.5)
Death	0 (0)	0 (0)
Worsening of glycemic control	4 (3.9)	10 (9.3)
Hypoglycemia	9 (8.8)	10 (9.3)
Myocardial infarction	1 (1.0)	0 (0)
Other coronary artery diseases	2 (2.0)	0 (0)
Stroke	0 (0)	1 (0.9)
Subarachnoid hemorrhage	0 (0)	1 (0.9)
Peripheral artery disease	1 (1.0)	0 (0)
Heart failure	0 (0)	1 (0.9)
Arrhythmia	2 (2.0)	0 (0)
Blood pressure reductions	1 (1.0)	0 (0)
Venous thrombosis	0 (0)	0 (0)
Volume depletion	1 (1.0)	0 (0)
Gastric cancer	1 (1.0)	0 (0)
Hepatic cancer	1 (1.0)	0 (0)
Prostate cancer	0 (0)	1 (0.9)
Brest cancer	1 (1.0)	1 (0.9)
Colon cancer	0 (0)	0 (0)
Malignant lymphoma	0 (0)	1 (0.9)
Anemia	0 (0)	0 (0)
Sleep apnea syndrome	0 (0)	1 (0.9)
Vertigo	2 (2.0)	0 (0)
Ophthalmic diseases	3 (2.9)	3 (2.8)
Otolaryngology disease	6 (5.9)	1 (0.9)
Dental diseases	2 (2.0)	0 (0)
Influenza, common cold	12 (11.8)	13 (12.0)
Pneumonia	0 (0)	1 (0.9)
Other respiratory disease	2 (2.0)	1 (0.9)
Epigastric discomfort	1 (1.0)	0 (0)
Digestive tract disease	9 (8.8)	8 (7.4)
Liver dysfunction	1 (1.0)	1 (0.9)
Renal dysfunction	0 (0)	2 (1.9)
Urinary lithiasis	1 (1.0)	1 (0.9)
Dyslipidemia	0 (0)	3 (2.8)
Thyroid disease	1 (1.0)	1 (0.9)
Urinary tract infection	4 (3.9)	2 (1.9)
Genital infection	2 (2.0)	0 (0)
Genital pruritus	1 (1.0)	0 (0)
Dermatitis	1 (1.0)	3 (2.8)
Eruption	6 (5.9)	6 (5.6)
Muscle spasm	2 (2.0)	1 (0.9)
Bone fracture	1 (1.0)	2 (1.9)
Other orthopedic disease	2 (2.0)	7 (6.5)
Edema	0 (0)	2 (1.9)
General fatigue	0 (0)	0 (0)
Traumatic injury	0 (0)	2 (1.9)
Others	8 (7.8)	14 (13.0)

Data are presented as numbers (%) of patients

## Discussion

In this study, we evaluated the effect of tofogliflozin on various health problems in patients with overweight or obesity with T2DM in real-life clinical practice. Our results showed that the tofogliflozin group achieved a weight loss of ≥ 3% at 26 weeks from baseline (46.4%; 95% CI 36.2–56.8%), which was approximately one in two patients and approximately three times higher than that in the conventional treatment group (14.4%; 95% CI 8.3–22.7%) (p < 0.001) (Fig. 2). Moreover, at 104 weeks, the percentage of patients achieving at least 3% weight loss was 62.0% (95% CI 51.2–71.9%) in the tofogliflozin group, which was significantly higher than that at 26 weeks, and significantly higher than that in the conventional treatment group (29.3%; 95% CI 20.6–39.3%) (p < 0.001). Thus, the weight loss effect of tofogliflozin administration was maintained for at least 2 years (Fig. 2).

Decrease in body water content due to diuresis contributes to weight loss under SGLT2 inhibitor treatment in the early phase of treatment; however, in the chronic phase, a decrease in body fat is known to be a major contributor to weight loss [23]. In fact, in the present study, waist circumference, one of the indicators of visceral fat accumulation, also decreased in the tofogliflozin group along with weight loss; furthermore, the degree of decrease in abdominal circumference was significantly greater in the tofogliflozin group than in the conventional treatment group. Therefore, the weight loss observed in the tofogliflozin group can be considered to reflect an improvement in obesity accompanied by fat loss.

Essentially, the goal of obesity treatment is to prevent and treat various obesity-related health problems. Obesity-related health disorders include glucose intolerance (including T2DM and impaired glucose tolerance), dyslipidemia, hypertension, hyperuricemia or gout, coronary artery disease, cerebral infarction or transient ischemic attack, nonalcoholic fatty liver disease (NAFLD), menstrual abnormalities or female infertility, obstructive sleep apnea syndrome or obesity hypoventilation syndrome, musculoskeletal disorders (including osteoarthritis of knee, hip, or finger joints as well as spondylosis deformans), and obesity-related kidney disease [19]. Therefore, in this study, we paid particular attention to glucose intolerance, dyslipidemia, hypertension, hyperuricemia or gout, coronary artery disease, cerebral vascular disease, NAFLD, and kidney disease, and evaluated the effect of tofogliflozin administration on the management status of these patients from the perspective of the percentage of achievement of management targets and change in the main management index.

First, with regard to the rate of achievement of control targets, particular attention was paid to HbA1c levels, blood pressure, LDL-C levels, triglyceride levels, HDL-C levels, and uric acid levels. Since the patients in this study had T2DM and obesity, we set management targets according to the treatment guidelines of the Japan Diabetes Society [24]. Hence, at week 26, the achievement rates of HbA1c (< 7%), systolic blood pressure (< 130 mmHg), and triglyceride levels (< 150 mg/dL) were significantly higher in the tofogliflozin group than in the conventional treatment group. The achievement rates of all the above factors also showed a tendency to be higher in the tofogliflozin group than in the conventional treatment group (Fig. 3 and Supplemental Table 2). The achievement rates of HDL-C (≥ 40 mg/dL) and uric acid (≤ 7 mg/dL) levels were around 90% in both treatment groups, with no significant difference observed between the two groups. At 104 weeks, the achievement rate of systolic blood pressure (< 130 mmHg) was significantly higher in the tofogliflozin group than in the conventional treatment group; however, there were no significant differences between the groups regarding the other items, with a slight increase in the achievement rate for each item in the conventional treatment group (Fig. 3 and Supplemental Table 2). It is assumed that the aggressive use of antihypertensive and dyslipidemic drugs in the conventional treatment group influenced this result. In contrast, only 9.2% (95% CI 4.1–17.3%) and 4.3% (95% CI 1.2–10.8%) of patients in the tofogliflozin and conventional treatment groups, respectively, achieved all outcome items, suggesting the need for more aggressive use of antihypertensive and dyslipidemic drugs.

Next, with regard to changes in control indices, HbA1c, fasting blood glucose, blood pressure, liver function-related indices (AST, ALT, γGTP), HDL-C, and serum uric acid were significantly improved in the tofogliflozin group at 26 weeks (Fig. 4 and Supplemental Table 3). The degree of improvement in these indices was significantly greater in the tofogliflozin group than in the conventional treatment group (Fig. 4 and Supplemental Table 3). Thus, improvement in obesity-related health problems was observed promptly after administration of tofogliflozin. Similar results were observed even after 104 weeks. Furthermore, the progression of arterial stiffness was significantly reduced (p < 0.05) and the increase in urinary albumin tended to be attenuated (p = 0.056) in the tofogliflozin group compared with the conventional treatment group. The improvement of QOL related to diabetes treatment, including treatment satisfaction regarding weight change, was also significantly greater in the tofogliflozin group than in the conventional treatment group.

Therefore, in patients with T2DM and overweight or obesity, administration of tofogliflozin was effective in improving obesity and had positive effects on obesity-related health problems including blood glucose levels, blood pressure, lipids, uric acid levels, liver function-related indicators, atherosclerosis, and QOL.

It has long been reported that SGLT2 inhibitors have positive effects on obesity and obesity-related health problems. In addition, through the main UTOPIA trial and its sub-analyses, we have previously reported that the administration of tofogliflozin, a relatively widely used SGLT2 inhibitor in Japan, in patients with T2DM showed beneficial effects on various cardiovascular risk factors including glycemic control, BMI, abdominal circumference, blood pressure, circulating levels of HDL-C, hepatic enzymes, and uric acid, suppressed the progression of atherosclerosis, and improved treatment-related QOL [12–15]. However, the UTOPIA study was not limited to patients with T2DM who were overweight or obese; although the mean BMI of the UTOPIA study participants was 27 kg/m^2^, which is slightly higher than the average BMI observed in Japanese patients with T2DM. Thus, the effects of tofogliflozin on obesity and obesity-related health problems have not been fully clarified. Therefore, to the best of our knowledge, this the first study to demonstrate the efficacy of tofogliflozin in overweight or obese Japanese patients with T2DM; hence, it may be of great benefit to daily clinical practice in Japan.

This study has several limitations. First, since the present study was a post-hoc analysis using data obtained from a clinical trial conducted for a different purpose, pre-specified studies may be warranted to confirm our findings. Furthermore, the open-label design of our study limited the evidence level of the study. Second, the administration of therapeutics other than tofogliflozin, including anti-diabetic and anti-hypertensive drugs may have affected the outcomes since these treatments were not matched completely. Third, the participants in this study were all Japanese patients with T2DM; hence, it may be premature to generalize our findings to other ethnic groups. Fourth, in this study, “obesity” was defined as a BMI of ≥ 25 kg/m^2^, with excess fat accumulation in adipose tissue, according to the definition of the JASSO Obesity Treatment Guidelines 2022. This threshold is lower than that of other guidelines such as those of the World Health Organization, the latter stipulating that individuals with a BMI of ≥ 25 kg/m^2^ but < 30 kg/m^2^ are overweight and those with a BMI of ≥ 30 kg/m^2^ are obese [25]. However, JASSO defines “obesity” as a BMI of ≥ 25 kg/m^2^ based on the finding that the mean number of obesity-related health disorders—including glucose intolerance, dyslipidemia, and hypertension—exceeded 1.0 when BMI reached 25 kg/m^2^ for Japanese individuals who underwent health checkups [26]. Since individuals of East Asian ethnicity, including Japanese, tend to develop certain obesity-related health disorders at a lower BMI than those of European ethnicity [27, 28], a lower BMI threshold is appropriate for the identification of Japanese individuals who are in need of medical intervention. Indeed, diagnosing obesity according to the JASSO criteria, the prevalence of certain types of obesity-related health disorders including T2DM and nonalcoholic steatohepatitis in Japan is similar to that in populations of European descent [27, 28]. Finally, among the obesity-related health disorders included in the JASSO diagnostic criteria for obesity (e.g. T2DM, dyslipidemia, hypertension, hyperuricemia or gout, coronary artery disease, cerebral infarction or transient ischemic attack, NAFLD, menstrual abnormalities or female infertility, obstructive sleep apnea syndrome or obesity hypoventilation syndrome, musculoskeletal disorders, and obesity-related kidney disease) [19], we were able to assess the management status of five (e.g. T2DM, dyslipidemia, hypertension, hyperuricemia, and NAFLD). However, we were unable to perform statistical evaluation of the incidence of coronary artery disease and cerebral infarction or transient ischemic attack due to the small number of events. In addition, we were unable to assess menstrual abnormalities or female infertility, obstructive sleep apnea syndrome or obesity hypoventilation syndrome, and musculoskeletal disorders because we did not collect sufficient information.

## Conclusions

In Japanese patients with T2DM and obesity, the SGLT2 inhibitor tofogliflozin is effective in improving obesity and has a positive impact on various obesity-related health problems (blood glucose levels, blood pressure, lipids, uric acid levels, liver function-related indicators, and atherosclerosis) and QOL. Future large scale studies in other ethnic groups are required to further validate our findings.

## Supplementary Information

Below is the link to the electronic supplementary material.

Supplementary file1 (DOCX 65 KB)

## Data Availability

The datasets generated and/or analyzed during our study will be available from the corresponding author upon reasonable request.
