# Supplementary material for: Effect of tofogliflozin on obesity-related health problems in patients with type 2 diabetes and overweight or obesity—a post-hoc sub-analysis of the UTOPIA study
Source: Diabetol Int. 2025 Aug 18;16(4):756–69. doi: 10.1007/s13340-025-00845-7 (PMC12532506; doi:10.1007/s13340-025-00845-7)
Supplement: Supplementary file 1 — Supplementary file1 (DOCX 65 KB) [file 13340_2025_845_MOESM1_ESM.docx]

**Supplementary Material 1**

**UTOPIA study investigators (listed in alphabetical order):**

*Hayashi Clinic:* I Hayashi

*Ikeda Municipal Hospital:* T Okada, M Tsugawa

*Jiyugaoka Medical Clinic:* H Yokoyama

*Juntendo Tokyo Koto Geriatric Medical Center:* H Yoshii

*Juntendo University Graduate School of Medicine:* T Mita, H Watada

*Kansai Rosai Hospital:* T Yamamoto

*Kanda Naika Clinic:* S Kawashima

*Kawasaki Hospital:* M Matsuda, T Nakamura

*Kawasaki Medical School:* H Kaneto

*Keio University School of Medicine:* Y Sato

*Kitasenri Maeda Clinic:* K Maeda

*Kosugi Medical Clinic:* K Kosugi

*Misaki Naika Clinic:* N Kuribayashi

*NakaKinen Clinic:* T Osonoi

*Nissay Hospital:* S Sumitani

*Osaka General Medical Center:* M Hatazaki, Y Umayahara

*National Hospital Organization Osaka National Hospital:* K Kato

*Osaka Police Hospital:* T Yasuda

*Osaka Rosai Hospital:* K Ryomoto

*Osaka University Graduate School of Medicine:* N Katakami, I Shimomura

*Otoshi Medical Clinic:* K Ohtoshi

*Shiraiwa Medical Clinic:* T Shiraiwa

*University of Occupational and Environmental Health, Japan:* A Kurozumi, Y Okada, K Torimoto

**UTOPIA trial site investigators (listed in alphabetical order):**

*Hayashi Clinic:* I Hayashi

*Ikeda Municipal Hospital:* M Tsugawa

*Jiyugaoka Medical Clinic:* H Yokoyama

*Juntendo Tokyo Koto Geriatric Medical Center:* H Yoshii

*Juntendo University Graduate School of Medicine:* K Komiyama, T Mita, T Shimizu

*Kansai Rosai Hospital:* T Yamamoto

*Kanda Naika Clinic:* S Kawashima

*Kawasaki Hospital:* T Nakamura

*Kawasaki Medical School:* S Kamei, T Kinoshita, M Shimoda

*Kitasenri Maeda Clinic:* K Maeda

*Kosugi Medical Clinic:* K Kosugi

*Misaki Naika Clinic:* H Yoshii

*NakaKinen Clinic:* H Ishida, T Osonoi, M Saito, A Tamazawa

*Nissay Hospital:* S Sumitani

*Osaka General Medical Center:* N Fujiki, Y Fujita, S Shimizu, Y Umayahara

*National Hospital Organization Osaka National Hospital:* K Kato

*Osaka Police Hospital:* Y Irie, R Kataoka, T Yasuda

*Osaka Rosai Hospital:* Y Kiyohara, M Ohashi, K Ryomoto, Y Takahi

*Osaka University Graduate School of Medicine:* Y Fujishima, Y Fujita, A Fukuhara, K Fukui, Y Hosokawa, A Imagawa, H Iwahashi, K Mukai, N Katakami, T Katsura, D Kawamori, T Kimura, S Kobayashi, J Kozawa, F Kubo, N Maeda, T Matsuoka,K Miyashita, S Nakata, H Ninomiya, H Nishizawa, Y Okuno, M Otsuki, F Sakamoto, S Sasaki, I Sato, N Shimo, I Shimomura, M Takahara, T Takano, A Tokunaga, S Uno, M Yamaoka, S Yoneda

*Otoshi Medical Clinic:* K Ohtoshi

*Shiraiwa Medical Clinic:* T Shiraiwa

*University of Occupational and Environmental Health, Japan:* M Hajime, K Koikawa, F Kuno, A Kurozumi, K Matsushita, M Narisawa, K Tanaka, K Sugai, Y Okada, K Torimoto

**Supplementary** **Material 2**

**Assessment of diabetes therapy-related QOL using Diabetes therapy-related QOL questionnaire (DTR-QOL) 7.**

The DTR-QOL developed by Ishii is a reliable and valid questionnaire, which is a 29-item, self-administered assessment with four primary factors, presented in Japanese [ref. S1]. In the current study, we used the DTR-QOL7, a short version of the original DTR-QOL, which consisted of 7 questions selected from original 29 items [ref. S2]. The items included are shown in Table S1. The response to each question consists of a 7-point Likert-type scale that ranges from 1 (strongly agree) to 7 (strongly disagree). The scales of Q5, Q6, and Q7 were reversed so that 7 represented the highest QOL score. The DTR-QOL7 was developed because of practical constraints, using data obtained from subjects with T2DM but without apparent history of CVD [ref. S2]. Although the method of selecting 7 questions from the original 29 items was not based on a technical or statistical rationale, we previously confirmed that all six items other than Q2 appeared to be included in the same domain, which suggested that the structure of the DTR-QOL7 was relatively consistent [ref. S2].

The total score, after simple addition of the item scores except the Q2 score, was converted to 0–100 (best-case response = 100; worst-case response = 0). This total score had a high internal consistency based on Cronbach's alpha coefficients, and they were highly associated with the total scores of original 29 items [ref. S2]. The Q2 score, which reflected weight gain with treatment, was separately evaluated. Each subject filled out the questionnaire and directly mailed it to the data center so that the researchers were blind to the answers. We treated the missing values according to the original DTR-QOL [ref. S1].

The DTR-QOL7 was evaluated at baseline, week 26, week 52, and week 104.

[ref. S1] Ishii H. Development and psychometric validation of the diabetes therapy-related QOL (DTR-QOL) questionnaire. J Med Econ. 2012;15:556–63.

[ref. S2] Mita T, Katakami N, Shiraiwa T, et al. The influence of sitagliptin on treatment-related quality of life in patients with type 2 diabetes mellitus receiving insulin treatment: A prespecified sub-analysis. Diabetes Ther. 2017;3;693–704.

**Table 1.** DTQ-QOL7 questionnaire.

| Q1. I am constantly concerned about time to manage my current diabetes treatment. |
| --- |
| Q2. I am bothered by weight gain with my current diabetes treatment. |
| Q3. I am sometimes bothered by low blood glucose. |
| Q4. I am worried about high blood glucose. |
| Q5. Overall, I am satisfied with my current blood sugar control. |
| Q6. With my current diabetes treatment, I am confident that I can maintain good blood glucose control. |
|  |
| Q7. With regard to diabetes treatment, I am satisfied with current treatment methods. |

**Supplementary Material 3**

**Safety evaluation**

All adverse events (AEs) were recorded during study. AEs were defined as any untoward medical occurrence in a clinical trial subject administered a medicinal product that were not necessarily related to this treatment. The details and incidence of all AEs were periodically ascertained. Based on the intention-to-treat the entire population, safety was checked by recording the AEs. When the investigators confirmed AEs, the severity grade, procedures, outcomes, and relationship to the study agent were assessed and reported to the trial organizer, the study secretariat, and the Data and Safety Monitoring Board (DSMB). The DSMB then deliberated on the incident and reported the decision to the chief investigator. Furthermore, serious AEs were reported to the principal investigator and the ethics committee. Both the investigator and the committee judged whether the diagnosis was appropriate or made a decision on whether the patient should be withdrawn from the trial. Cardiovascular events were diagnosed and fully assessed by members of the Cardiovascular Endpoint committee (which included two cardiologists and a neurologist).

**Supplemental Table 1. Principal concomitant medication use during the observation period.**

| **Parameters** | **Tofogliflozin treatment group** | **Conventional treatment group** | **P value** |
| --- | --- | --- | --- |
| Use of glucose-lowering agents* |  |  |  |
| Baseline | 93 (91.2) | 97 (89.8) | 0.816 |
| Week 26 | 91 (91.0) | 99 (92.5) | 0.802 |
| Week 52 | 86 (89.6) | 97 (92.4) | 0.622 |
| Week 78 | 83 (89.2) | 96 (93.2) | 0.447 |
| Week 104 | 83 (89.2) | 93 (93.0) | 0.449 |
| Metformin |  |  |  |
| Baseline | 64 (62.7) | 65 (60.2) | 0.777 |
| Week 26 | 63 (63.0) | 68 (63.6) | 1.000 |
| Week 52 | 59 (61.5) | 68 (64.8) | 0.662 |
| Week 78 | 56 (60.2) | 67 (65.0) | 0.554 |
| Week 104 | 57 (61.3) | 66 (66.0) | 0.550 |
| Sulfonylurea |  |  |  |
| Baseline | 21 (20.6) | 24 (22.2) | 0.867 |
| Week 26 | 19 (19.0) | 24 (22.4) | 0.609 |
| Week 52 | 20 (20.8) | 27 (25.7) | 0.505 |
| Week 78 | 19 (20.4) | 22 (21.4) | 1.000 |
| Week 104 | 18 (19.4) | 21 (21.0) | 0.858 |
| Glinides |  |  |  |
| Baseline | 6 (5.9) | 4 (3.7) | 0.529 |
| Week 26 | 6 (6.0) | 4 (3.7) | 0.527 |
| Week 52 | 5 (5.2) | 4 (3.8) | 0.739 |
| Week 78 | 5 (5.4) | 4 (3.9) | 0.738 |
| Week 104 | 5 (5.4) | 3 (3.0) | 0.485 |
| Thiazolidinediones |  |  |  |
| Baseline | 15 (14.7) | 15 (13.9) | 1.000 |
| Week 26 | 14 (14.0) | 15 (14.0) | 1.000 |
| Week 52 | 15 (15.6) | 16 (15.2) | 1.000 |
| Week 78 | 15 (16.1) | 14 (13.6) | 0.689 |
| Week 104 | 15 (16.1) | 12 (12.0) | 0.534 |
| α-glucosidase inhibitors |  |  |  |
| Baseline | 12 (11.8) | 12 (11.1) | 1.000 |
| Week 26 | 13 (13.0) | 14 (13.1) | 1.000 |
| Week 52 | 11 (11.5) | 14 (13.3) | 0.831 |
| Week 78 | 11 (11.8) | 14 (13.6) | 0.831 |
| Week 104 | 11 (11.8) | 15 (15.0) | 0.536 |
| DPP-4 inhibitors |  |  |  |
| Baseline | 41 (40.2) | 58 (53.7) | 0.054 |
| Week 26 | 38 (38.0) | 59 (55.1) | 0.018 |
| Week 52 | 38 (39.6) | 58 (55.2) | 0.034 |
| Week 78 | 37 (39.8) | 57 (55.3) | 0.033 |
| Week 104 | 35 (37.6) | 56 (56.0) | 0.014 |
| GLP-1 R agonists |  |  |  |
| Baseline | 17 (16.7) | 11 (10.2) | 0.223 |
| Week 26 | 17 (17.0) | 13 (12.1) | 0.332 |
| Week 52 | 17 (17.7) | 14 (13.3) | 0.438 |
| Week 78 | 16 (17.2) | 12 (11.7) | 0.310 |
| Week 104 | 17 (18.3) | 11 (11.0) | 0.160 |
| Insulins |  |  |  |
| Baseline | 20 (19.6) | 27 (25.0) | 0.409 |
| Week 26 | 18 (18.0) | 27 (25.2) | 0.240 |
| Week 52 | 17 (17.7) | 29 (27.6) | 0.130 |
| Week 78 | 15 (16.1) | 29 (28.2) | 0.059 |
| Week 104 | 15 (16.1) | 27 (27.0) | 0.081 |
|  |  |  |  |
| Use of any antihypertensive drugs |  |  |  |
| Baseline | 54 (52.9) | 67 (62.0) | 0.209 |
| Week 26 | 53 (53.0) | 70 (65.4) | 0.089 |
| Week 52 | 51 (53.1) | 70 (66.7) | 0.061 |
| Week 78 | 48 (51.6) | 69 (67.0) | 0.030 |
| Week 104 | 48 (51.6) | 66 (66.0) | 0.057 |
| Angiotensin-converting enzyme inhibitors |  |  |  |
| Baseline | 3 (2.9) | 3 (2.8) | 1.000 |
| Week 26 | 3 (3.0) | 3 (2.8) | 1.000 |
| Week 52 | 2 (2.1) | 3 (2.9) | 1.000 |
| Week 78 | 2 (2.2) | 3 (2.9) | 1.000 |
| Week 104 | 2 (2.2) | 3 (3.0) | 1.000 |
| Angiotensin II receptor blockers |  |  |  |
| Baseline | 46 (45.1) | 59 (54.6) | 0.214 |
| Week 26 | 45 (45.0) | 61 (57.0) | 0.096 |
| Week 52 | 44 (45.8) | 60 (57.1) | 0.122 |
| Week 78 | 41 (44.1) | 62 (60.2) | 0.032 |
| Week 104 | 40 (43.0) | 59 (59.0) | 0.031 |
| Calcium channel blockers |  |  |  |
| Baseline | 33 (32.4) | 39 (36.1) | 0.663 |
| Week 26 | 32 (32.0) | 40 (37.4) | 0.466 |
| Week 52 | 32 (33.3) | 40 (38.1) | 0.556 |
| Week 78 | 31 (33.3) | 41 (39.8) | 0.376 |
| Week 104 | 32 (34.4) | 40 (40.0) | 0.458 |
|  |  |  |  |
| Use of any lipid-lowering agents |  |  |  |
| Baseline | 55 (53.9) | 68 (63.0) | 0.208 |
| Week 26 | 54 (54.0) | 68 (63.6) | 0.203 |
| Week 52 | 51 (53.1) | 69 (65.7) | 0.084 |
| Week 78 | 54 (58.1) | 68 (66.0) | 0.302 |
| Week 104 | 53 (57.0) | 67 (67.0) | 0.182 |
| Statins |  |  |  |
| Baseline | 50 (49.0) | 54 (50.0) | 0.891 |
| Week 26 | 49 (49.0) | 54 (50.5) | 0.890 |
| Week 52 | 46 (47.9) | 56 (53.3) | 0.482 |
| Week 78 | 48 (51.6) | 57 (55.3) | 0.668 |
| Week 104 | 48 (51.6) | 56 (56.0) | 0.566 |
|  |  |  |  |
| Use of any antithrombotic agents |  |  |  |
| Baseline | 9 (8.8) | 11 (10.2) | 0.816 |
| Week 26 | 9 (9.0) | 10 (9.3) | 1.000 |
| Week 52 | 9 (9.4) | 11 (10.5) | 0.818 |
| Week 78 | 8 (8.6) | 11 (10.7) | 0.810 |
| Week 104 | 8 (8.6) | 10 (10.0) | 0.808 |
| Antiplatelet drugs |  |  |  |
| Baseline | 8 (7.8) | 8 (7.4) | 1.000 |
| Week 26 | 8 (8.0) | 7 (6.5) | 0.791 |
| Week 52 | 8 (8.3) | 8 (7.6) | 1.000 |
| Week 78 | 7 (7.5) | 8 (7.8) | 1.000 |
| Week 104 | 7 (7.5) | 8 (8.0) | 1.000 |
| Anticoagulants |  |  |  |
| Baseline | 1 (1.0) | 3 (2.8) | 0.622 |
| Week 26 | 1 (1.0) | 3 (2.8) | 0.622 |
| Week 52 | 1 (1.0) | 3 (2.9) | 0.623 |
| Week 78 | 1 (1.1) | 3 (2.9) | 0.623 |
| Week 104 | 1 (1.1) | 2 (2.0) | 1.000 |

Data are presented as number (%) of patients. Differences in parameters between groups were analyzed using Fisher's exact test.

*; Administration of tofogliflozin in the tofogliflozin treatment group was not counted as concomitantly used anti-diabetic agents.

**Supplemental Table 2. Percentage of treatment goal achieved in both treatment groups at each observation point and the difference between treatment groups**

| Parameters | Tofogliflozin group | Conventional group | P value |
| --- | --- | --- | --- |
| HbA1c < 7% at baseline (%) | 30.4 [21.7–40.3] (n=102) | 30.6 [22.1–40.2] (n=108) | 0.979 |
| Week 26 | 52.0 [41.7–62.2] (n=98) | 28.8 [20.4–38.6] (n=104) | 0.001 |
| Week 52 | 47.4 [37.2–57.8] (n=97) | 34.6 [25.6–44.6] (n=104) | 0.065 |
| Week 78 | 50.5 [39.9–61.2] (n=91) | 39.6 [30.0–49.8] (n=101) | 0.128 |
| Week 104 | 47.3 [36.9–57.9] (n=93) | 37.1 [27.5–47.5] (n=97) | 0.155 |
| Systolic blood pressure < 130 mmHg at baseline (%) | 39.0 [29.4–49.3] (n=100) | 43.4 [33.8–53.4] (n=106) | 0.522 |
| Week 26 | 55.1 [44.7–65.2] (n=98) | 35.9 [26.7–46.0] (n=103) | 0.006 |
| Week 52 | 61.9 [51.4–71.5] (n=97) | 47.6 [37.6–57.6] (n=103) | 0.043 |
| Week 78 | 48.9 [38.3–59.6] (n=92) | 42.0 [32.2–52.3] (n=100) | 0.336 |
| Week 104 | 57.1 [46.3–67.5] (n=91) | 35.7 [26.3–46.0] (n=98) | 0.003 |
| Diastolic blood pressure < 80 mmHg at baseline (%) | 47.0 [36.9–57.2] (n=100) | 45.3 [35.6–55.2] (n=106) | 0.805 |
| Week 26 | 56.1 [45.7–66.1] (n=98) | 46.6 [36.7–56.7] (n=103) | 0.177 |
| Week 52 | 62.9 [52.5–72.5] (n=97) | 53.4 [43.3–63.3] (n=103) | 0.174 |
| Week 78 | 62.0 [51.2–71.9] (n=92) | 48.0 [37.9–58.2] (n=100) | 0.052 |
| Week 104 | 64.8 [54.1–74.6] (n=91) | 51.0 [40.7–61.3] (n=98) | 0.055 |
| LDL-C < 120 mg/dL at baseline (%) | 67.6 [57.7–76.6] (n=102) | 59.8 [49.9–69.2] (n=107) | 0.239 |
| Week 26 | 68.8 [58.5–77.8] (n=96) | 62.4 [52.2–71.8] (n=101) | 0.347 |
| Week 52 | 63.5 [53.1–73.1] (n=96) | 68.3 [58.4–77.1] (n=104) | 0.481 |
| Week 78 | 64.8 [54.1–74.6] (n=91) | 68.7 [58.6–77.6] (n=99) | 0.573 |
| Week 104 | 65.2 [54.6–74.9] (n=92) | 63.3 [52.9–72.8] (n=98) | 0.779 |
| Triglyceride < 150 mg/dL at baseline (%) | 67.6 [57.7–76.6] (n=102) | 55. [45.2–64.8] (n=107) | 0.064 |
| Week 26 | 71.4 [61.0–80.4] (n=91) | 57.4 [46.8–67.6] (n=94) | 0.047 |
| Week 52 | 72.2 [61.8–81.1] (n=90) | 67.0 [56.9–76.1] (n=100) | 0.435 |
| Week 78 | 67.1 [56.0–76.9] (n=85) | 64.8 [54.1–74.6] (n=91) | 0.756 |
| Week 104 | 66.7 [55.9–76.3] (n=90) | 68.8 [58.4–78.0] (n=93) | 0.756 |
| HDL-C ≥ 40 mg/dL at baseline (%) | 84.3 [75.8–90.8] (n=102) | 88.0 [80.3–93.4] (n=108) | 0.444 |
| Week 26 | 92.7 [85.6–97.0] (n=96) | 86.3 [78.0–92.3] (n=102) | 0.142 |
| Week 52 | 88.7 [80.6–94.2] (n=97) | 83.7 [75.1–90.2] (n=104) | 0.306 |
| Week 78 | 93.4 [86.2–97.5] (n=91) | 87.1 [79.0–93.0] (n=101) | 0.146 |
| Week 104 | 93.5 [86.5–97.6] (n=93) | 88.8 [80.0–94.3] (n=98) | 0.247 |
| Uric acid ≤ 7 mg/dL at baseline (%) | 92.1 [85.0–96.5] (n=101) | 87.9 [80.1–93.4] (n=107) | 0.312 |
| Week 26 | 93.6 [86.6–97.6] (n=94) | 92.0 [84.8–96.5] (n=100) | 0.664 |
| Week 52 | 93.7 [86.8–97.6] (n=95) | 92.3 [85.4–96.6] (n=104) | 0.704 |
| Week 78 | 95.6 [89.0–98.8] (n=90) | 91.9 [84.7–96.4] (n=99) | 0.379 |
| Week 104 | 92.5 [85.1–96.9] (n=93) | 90.8 [83.3–95.7] (n=98) | 0.680 |
| Achievement of seven goals above at baseline (%) | 3.0 [0.6–8.6] (n=99) | 4.9 [1.6–11.0] (n=103) | 0.721 |
| Week 26 | 9.0 [4.0–16.9] (n=89) | 2.2 [0.3–7.6] (n=92) | 0.055 |
| Week 52 | 10.1 [4.7–18.3] (n=89) | 5.0 [1.6–11.3] (n=100) | 0.266 |
| Week 78 | 6.0 [2.0–13.3] (n=84) | 2.2 [0.3–7.7] (n=91) | 0.263 |
| Week 104 | 9.2 [4.1–17.3] (n=87) | 4.3 [1.2–10.8] (n=92) | 0.240 |

Treatment goal achievement rates are expressed as percentage (95% confidence interval).

HbA1c, glycated hemoglobin; LDL-C, low-density lipoprotein-cholesterol; HDL-C, high-density lipoprotein-cholesterol; eGFR, estimated glomerular filtration rate.

**Supplemental Table 3. Changes over time and within- and between-group comparisons of clinical parameters at each observation point**

| Parameters | Tofogliflozin group | Conventional group | P value |
| --- | --- | --- | --- |
| Body weight at baseline (kg) | 79.8±15.9 (n=102) | 80.2±12.9 (n=108) | 0.838 |
| Week 26 (change from baseline) | -2.0±3.2 (n=97)§ | -0.6±2.2 (n=104)* | < 0.001 |
| Week 52 (change from baseline) | -2.4±3.9 (n=97)§ | -0.9±2.7 (n=102)# | 0.002 |
| Week 78 (change from baseline) | -2.5±4.8 (n=92)§ | -0.7±3.7 (n=100) | 0.006 |
| Week 104 (change from baseline) | -2.8±4.5 (n=92)§ | -1.4±4.5 (n=99)# | 0.032 |
| Body Mass Index at baseline (kg/m^2^) | 30.0±5.4 (n=102) | 29.7±3.4 (n=108) | 0.616 |
| Week 26 (change from baseline) | -0.8±1.2 (n=97)§ | -0.2±0.8 (n=104)* | < 0.001 |
| Week 52 (change from baseline) | -0.9±1.5 (n=97)§ | -0.3±1.0 (n=102)# | 0.002 |
| Week 78 (change from baseline) | -0.9±1.8 (n=92)§ | -0.3±1.3 (n=100)* | 0.005 |
| Week 104 (change from baseline) | -1.0±1.7 (n=92)§ | -0.5±1.6 (n=99)# | 0.026 |
| Waist circumference at baseline (cm) | 99.9±10.8 (n=90) | 99.4±9.8 (n=98) | 0.745 |
| Week 26 (change from baseline) | -2.6±6.7 (n=74)# | 0.9±4.2 (n=81) | < 0.001 |
| Week 52 (change from baseline) | -0.6±6.9 (n=75) | 1.1±5.2 (n=84) | 0.087 |
| Week 78 (change from baseline) | -1.9±7.2 (n=66)* | 0.8±4.7 (n=76) | 0.010 |
| Week 104 (change from baseline) | -0.9±6.8 (n=72) | 1.1±4.2 (n=77)* | 0.031 |
| HbA1c at baseline (%) | 7.5±0.8 (n=102) | 7.4±0.8 (n=108) | 0.449 |
| Week 26 (change from baseline) | -0.5±0.6 (n=98)§ | 0.0±0.6 (n=104) | < 0.001 |
| Week 52 (change from baseline) | -0.4±0.7 (n=97)§ | -0.1±0.6 (n=104) | < 0.001 |
| Week 78 (change from baseline) | -0.4±0.8 (n=91)§ | -0.1±0.6 (n=101) | 0.002 |
| Week 104 (change from baseline) | -0.4±0.8 (n=93)§ | 0.0±0.8 (n=97) | < 0.001 |
| Fasting blood glucose at baseline (mg/dL) | 144.7±33.0 (n=102) | 143.0±34.7 (n=107) | 0.714 |
| Week 26 (change from baseline) | -19.6±32.8 (n=93)§ | 5.2±42.4 (n=95) | < 0.001 |
| Week 52 (change from baseline) | -17.9±32.7 (n=90)§ | -1.3±33.2 (n=99) | < 0.001 |
| Week 78 (change from baseline) | -12.2±30.3 (n=85)§ | 1.6±37.0 (n=91) | 0.007 |
| Week 104 (change from baseline) | -15.3±32.5 (n=90)§ | 2.8±33.8 (n=94) | < 0.001 |
| Systolic blood pressure at baseline (mmHg) | 134.0±14.0 (n=100) | 135.5±17.5 (n=106) | 0.494 |
| Week 26 (change from baseline) | -5.3±13.9 (n=96)§ | -0.1±15.7 (n=101) | 0.014 |
| Week 52 (change from baseline) | -6.9±14.4 (n=95)§ | -3.8±17.5 (n=102)* | 0.173 |
| Week 78 (change from baseline) | -5.6±16.1 (n=90)# | -2.1±17.0 (n=98) | 0.151 |
| Week 104 (change from baseline) | -6.1±17.6 (n=89)# | 0.7±16.8 (n=96) | 0.008 |
| Diastolic blood pressure at baseline (mmHg) | 78.8±9.5 (n=100) | 80.6±10.7 (n=106) | 0.189 |
| Week 26 (change from baseline) | -1.5±9.0 (n=96) | -0.2±9.4 (n=101) | 0.337 |
| Week 52 (change from baseline) | -3.8±9.6 (n=95)§ | -1.5±10.6 (n=102) | 0.115 |
| Week 78 (change from baseline) | -2.5±9.1 (n=90)* | -1.0±10.1 (n=98) | 0.296 |
| Week 104 (change from baseline) | -3.1±10.0 (n=89)# | -0.9±10.1 (n=96) | 0.131 |
| AST at baseline (IU/L) | 28.2±12.3 (n=102) | 29.2±16.6 (n=108) | 0.630 |
| Week 26 (change from baseline) | -4.3±8.9 (n=96)§ | -0.4±9.8 (n=102) | 0.004 |
| Week 52 (change from baseline) | -5.1±9.9 (n=97)§ | -3.4±12.9 (n=104)# | 0.295 |
| Week 78 (change from baseline) | -3.5±12.2 (n=92)# | -0.7±21.3 (n=101) | 0.261 |
| Week 104 (change from baseline) | -4.2±10.2 (n=93)§ | -2.6±13.3 (n=98) | 0.350 |
| ALT at baseline (IU/L) | 35.4±23.4 (n=102) | 35.1±28.2 (n=108) | 0.934 |
| Week 26 (change from baseline) | -8.9±15.4 (n=96)§ | -1.6±12.7 (n=102) | < 0.001 |
| Week 52 (change from baseline) | -9.5±17.2 (n=97)§ | -5.9±19.8 (n=104)# | 0.179 |
| Week 78 (change from baseline) | -6.7±20.7 (n=92)# | -4.2±23.4 (n=101) | 0.425 |
| Week 104 (change from baseline) | -8.6±17.8 (n=93)§ | -4.0±22.5 (n=98) | 0.117 |
| γ-GTP at baseline (U/L) | 45.3±37.2 (n=100) | 43.2±32.5 (n=108) | 0.673 |
| Week 26 (change from baseline) | -7.4±21.0 (n=95)§ | 0.0±18.4 (n=101) | 0.010 |
| Week 52 (change from baseline) | -6.5±22.5 (n=96)# | -3.7±21.7 (n=104) | 0.366 |
| Week 78 (change from baseline) | -4.4±38.5 (n=91) | 0.5±30.3 (n=100) | 0.339 |
| Week 104 (change from baseline) | -9.4±25.5 (n=92)§ | -3.3±20.1 (n=97) | 0.068 |
| Total cholesterol at baseline (mg/dL) | 187.5±28.0 (n=100) | 190.1±32.2 (n=103) | 0.540 |
| Week 26 (change from baseline) | 1.2±20.2 (n=91) | -0.8±21.4 (n=95) | 0.520 |
| Week 52 (change from baseline) | 2.7±23.0 (n=94) | -3.5±22.0 (n=100) | 0.056 |
| Week 78 (change from baseline) | 3.2±21.5 (n=84) | -4.0±26.6 (n=92) | 0.048 |
| Week 104 (change from baseline) | 2.5±27.3 (n=90) | 0.1±24.6 (n=94) | 0.537 |
| LDL-C at baseline (mg/dL) | 109.6±25.1 (n=102) | 112.8±25.1 (n=107) | 0.361 |
| Week 26 (change from baseline) | -0.2±18.7 (n=96) | -0.4±20.4 (n=101) | 0.932 |
| Week 52 (change from baseline) | 0.5±19.5 (n=96) | -2.4±18.8 (n=104) | 0.286 |
| Week 78 (change from baseline) | 1.2±19.4 (n=91) | -4.8±23.8 (n=99)* | 0.057 |
| Week 104 (change from baseline) | -0.7±26.0 (n=92) | -2.2±19.9 (n=98) | 0.664 |
| HDL-C at baseline (mg/dL) | 51.8±12.3 (n=102) | 50.8±11.6 (n=108) | 0.522 |
| Week 26 (change from baseline) | 2.2±6.5 (n=96)# | 0.0±6.9 (n=102) | 0.020 |
| Week 52 (change from baseline) | 2.1±6.1 (n=97)§ | 0.1±6.9 (n=104) | 0.028 |
| Week 78 (change from baseline) | 2.5±6.9 (n=91)§ | 0.7±7.7 (n=101) | 0.094 |
| Week 104 (change from baseline) | 2.2±6.5 (n=93)# | 2.5±7.7 (n=98)# | 0.766 |
| Triglyceride at baseline (mg/dL) | 142.9±82.0 (n=102) | 151.7±71.8 (n=107) | 0.410 |
| Week 26 (change from baseline) | -9.7±61.7 (n=91) | 0.8±51.0 (n=93) | 0.210 |
| Week 52 (change from baseline) | -1.9±60.9 (n=90) | -13.8±63.1 (n=99)* | 0.191 |
| Week 78 (change from baseline) | 7.7±85.8 (n=85) | 4.3±121.4 (n=90) | 0.832 |
| Week 104 (change from baseline) | 0.7±55.8 (n=90) | -2.3±81.9 (n=93) | 0.774 |
| Uric acid at baseline (mg/dL) | 5.7±1.2 (n=101) | 5.6±1.2 (n=107) | 0.650 |
| Week 26 (change from baseline) | -0.4±0.8 (n=93)§ | -0.1±0.7 (n=100) | 0.006 |
| Week 52 (change from baseline) | -0.4±0.9 (n=95)§ | -0.1±0.7 (n=103) | 0.003 |
| Week 78 (change from baseline) | -0.5±0.8 (n=90)§ | -0.1±0.8 (n=99) | < 0.001 |
| Week 104 (change from baseline) | -0.4±0.9 (n=92)§ | -0.1±0.8 (n=97) | 0.015 |
| eGFR at baseline (mL/min/1.73 m^2^) | 81.0±20.6 (n=101) | 82.4±24.6 (n=107) | 0.644 |
| Week 26 (change from baseline) | -3.9±9.4 (n=94)§ | -2.4±9.4 (n=101)* | 0.266 |
| Week 52 (change from baseline) | -4.4±8.9 (n=95)§ | -2.6±9.3 (n=103)# | 0.147 |
| Week 78 (change from baseline) | -4.1±10.1 (n=91)§ | -3.7±11.1 (n=100)# | 0.780 |
| Week 104 (change from baseline) | -4.3±11.0 (n=92)§ | -3.3±9.9 (n=97)# | 0.519 |
| Urinary albumin excretion at baseline (mg/g/cre) | 68.1±159.8 (n=94) | 134±378 (n=101) | 0.110 |
| Week 26 (change from baseline) | -11.97±130.6 (n=78) | 6.1±177.6 (n=87) | 0.455 |
| Week 52 (change from baseline) | -22.9±109.2 (n=84) | 0.2±131.6 (n=93) | 0.203 |
| Week 78 (change from baseline) | 93.2±935.9 (n=74) | 54.8±317.9 (n=84) | 0.737 |
| Week 104 (change from baseline) | -12.7±193.7 (n=85) | 56.6±275.7 (n=89) | 0.056 |
| White blood cell count at baseline (/μL) | 6965.2±1991.6 (n=100) | 6789.7±1997.7 (n=108) | 0.527 |
| Week 26 (change from baseline) | 326.6±1214.4 (n=92)* | 133.3±1480.5 (n=97) | 0.327 |
| Week 52 (change from baseline) | 216.7±1686.6 (n=95) | -66.1±1496.8 (n=101) | 0.217 |
| Week 78 (change from baseline) | 49.6±1397.9 (n=90) | 216.9±1881.5 (n=95) | 0.492 |
| Week 104 (change from baseline) | -44.3±1667.9 (n=91) | 110.2±1555.4 (n=95) | 0.515 |
| Red blood cell count at baseline (×10^4^/μL) | 472.3±47.4 (n=100) | 476.5±47.1 (n=108) | 0.515 |
| Week 26 (change from baseline) | 21.9±24.8 (n=92)§ | 0.4±24.6 (n=97) | < 0.001 |
| Week 52 (change from baseline) | 20.2±35.0 (n=95)§ | -2.5±27.8 (n=101) | < 0.001 |
| Week 78 (change from baseline) | 21.0±34.7 (n=90)§ | -8.4±37.0 (n=95)* | < 0.001 |
| Week 104 (change from baseline) | 20.9±30.7 (n=91)§ | -0.9±25.8 (n=95) | < 0.001 |
| Hemoglobin at baseline (g/dL) | 14.3±1.6 (n=100) | 14.3±1.4 (n=108) | 0.898 |
| Week 26 (change from baseline) | 0.6±0.8 (n=92)§ | 0.0±0.7 (n=97) | < 0.001 |
| Week 52 (change from baseline) | 0.6±1.2 (n=95)§ | -0.1±0.9 (n=101) | < 0.001 |
| Week 78 (change from baseline) | 0.8±1.2 (n=90)§ | -0.3±1.2 (n=95)* | < 0.001 |
| Week 104 (change from baseline) | 0.7±1.2 (n=91)§ | 0.0±0.9 (n=95) | < 0.001 |
| Hematocrit at baseline (%) | 43.0±4.4 (n=100) | 43.1±3.9 (n=108) | 0.927 |
| Week 26 (change from baseline) | 2.1±2.3 (n=92)§ | -0.1±2.3 (n=97) | < 0.001 |
| Week 52 (change from baseline) | 2.0±3.6 (n=95)§ | -0.4±2.6 (n=101) | < 0.001 |
| Week 78 (change from baseline) | 2.3±3.5 (n=90)§ | -0.9±3.7 (n=95)* | < 0.001 |
| Week 104 (change from baseline) | 2.3±3.4 (n=91)§ | -0.2±2.8 (n=95) | < 0.001 |
| Platelet count at baseline (×10^4^/μL) | 23.2±5.4 (n=99) | 23.4±5.8 (n=108) | 0.756 |
| Week 26 (change from baseline) | 0.1±2.9 (n=91) | 0.1±3.0 (n=97) | 0.934 |
| Week 52 (change from baseline) | 0.0±3.1 (n=94) | 0.4±3.6 (n=101) | 0.408 |
| Week 78 (change from baseline) | -0.6±2.8 (n=89) | 0.2±3.9 (n=95) | 0.129 |
| Week 104 (change from baseline) | 0.0±2.9 (n=90) | 0.2±4.1 (n=95) | 0.744 |
| Adiponectin at baseline (μg/mL) | 8.1±5.4 (n=102) | 8.8±6.9 (n=108) | 0.368 |
| Week 52 (change from baseline) | 0.9±2.5 (n=92)§ | 0.6±1.6 (n=101) | 0.308 |
| Week 104 (change from baseline) | 1.1±2.9 (n=91)§ | 0.4±3.2 (n=96) | 0.104 |
| NT-proBNP at baseline (pg/mL) | 51.5±106.8 (n=102) | 98.8±304.9 (n=108) | 0.131 |
| Week 52 (change from baseline) | -1.2±52.4 (n=92) | 64.2±585.5 (n=101) | 0.266 |
| Week 104 (change from baseline) | -4.2±65.6 (n=91) | 96.7±802.4 (n=96) | 0.222 |
| Right maximum CCA-IMT at baseline (mm) | 1.05±0.22 (n=102) | 1.06±0.26 (n=108) | 0.921 |
| Week 52 (change from baseline) | -0.119±0.150 (n=92)§ | -0.123±0.147 (n=102)§ | 0.865 |
| Week 104 (change from baseline) | -0.173±0.154 (n=90)§ | -0.200±0.161 (n=93)§ | 0.256 |
| Left maximum CCA-IMT at baseline (mm) | 1.12±0.35 (n=102) | 1.08±0.35 (n=108) | 0.431 |
| Week 52 (change from baseline) | -0.129±0.273 (n=92)§ | -0.112±0.159 (n=102)§ | 0.607 |
| Week 104 (change from baseline) | -0.178±0.259 (n=90)§ | -0.188±0.161 (n=93)§ | 0.761 |
| Right mean CCA-IMT at baseline (mm) | 0.85±0.14 (n=102) | 0.84±0.16 (n=108) | 0.721 |
| Week 52 (change from baseline) | -0.080±0.082 (n=92)§ | -0.088±0.074 (n=102)§ | 0.496 |
| Week 104 (change from baseline) | -0.133±0.092 (n=90)§ | -0.141±0.087 (n=93)§ | 0.576 |
| Left mean CCA-IMT at baseline (mm) | 0.89±0.21 (n=102) | 0.87±0.21 (n=108) | 0.533 |
| Week 52 (change from baseline) | -0.106±0.096 (n=92)§ | -0.097±0.091 (n=102)§ | 0.506 |
| Week 104 (change from baseline) | -0.156±0.137 (n=90)§ | -0.161±0.096 (n=93)§ | 0.786 |
| Mean CCA-IMT at baseline (mm) | 0.87±0.16 (n=102) | 0.86±0.17 (n=108) | 0.570 |
| Week 52 (change from baseline) | -0.093±0.070 (n=92)§ | -0.093±0.067 (n=102)§ | 0.947 |
| Week 104 (change from baseline) | -0.145±0.098 (n=90)§ | -0.151±0.076 (n=93)§ | 0.639 |
| Right maximum BIF-IMT at baseline (mm) | 1.54±0.65 (n=84) | 1.48±0.71 (n=94) | 0.531 |
| Week 52 (change from baseline) | -0.214±0.527 (n=76)§ | -0.254±0.348 (n=89)§ | 0.577 |
| Week 104 (change from baseline) | -0.405±0.404 (n=75)§ | -0.351±0.342 (n=80)§ | 0.374 |
| Left maximum BIF-IMT at baseline (mm) | 1.42±0.59 (n=86) | 1.40±0.62 (n=95) | 0.893 |
| Week 52 (change from baseline) | -0.176±0.250 (n=75)§ | -0.203±0.317 (n=90)§ | 0.541 |
| Week 104 (change from baseline) | -0.255±0.303 (n=73)§ | -0.296±0.356 (n=83)§ | 0.443 |
| Right maximum ICA-IMT at baseline (mm) | 1.02±0.38 (n=46) | 1.00±0.55 (n=56) | 0.823 |
| Week 52 (change from baseline) | -0.138±0.192 (n=38)§ | -0.157±0.258 (n=48)§ | 0.698 |
| Week 104 (change from baseline) | -0.235±0.214 (n=35)§ | -0.184±0.411 (n=39)# | 0.506 |
| Left maximum ICA-IMT at baseline (mm) | 0.92±0.39 (n=50) | 0.98±0.47 (n=55) | 0.525 |
| Week 52 (change from baseline) | -0.097±0.150 (n=41)§ | -0.169±0.400 (n=49)# | 0.251 |
| Week 104 (change from baseline) | -0.130±0.202 (n=38)§ | -0.249±0.402 (n=40)§ | 0.103 |
| Right baPWV at baseline (cm/s) | 1654.4±431.4 (n=46) | 1669.2±274.3 (n=42) | 0.847 |
| Week 52 (change from baseline) | -55.6±243.7 (n=42) | 26.4±191.6 (n=37) | 0.099 |
| Week 104 (change from baseline) | -90.0±296.3 (n=37) | 74.1±213.7 (n=34) | 0.009 |
| Left baPWV at baseline (cm/s) | 1680.5±495.0 (n=46) | 1664.3±303.0 (n=42) | 0.852 |
| Week 52 (change from baseline) | -75.5±237.3 (n=42)* | 25.2±193.4 (n=37) | 0.041 |
| Week 104 (change from baseline) | -70.7±268.7 (n=37) | 93.9±256.9 (n=34)* | 0.010 |
| mean baPWV at baseline (cm/s) | 1667.4±455.8 (n=46) | 1666.8±280.1 (n=42) | 0.993 |
| Week 52 (change from baseline) | -65.5±230.5 (n=42) | 25.8±181.5 (n=37) | 0.053 |
| Week 104 (change from baseline) | -80.4±275.5 (n=37) | 84.0±221.8 (n=34)* | 0.007 |
| Right ABI at baseline | 1.13±0.11 (n=62) | 1.14±0.11 (n=66) | 0.502 |
| Week 52 (change from baseline) | 0.00±0.10 (n=53) | 0.00±0.10 (n=53) | 0.576 |
| Week 104 (change from baseline) | 0.00±0.10 (n=47) | 0.00±0.09 (n=47) | 0.574 |
| Left ABI at baseline | 1.10±0.10 (n=61) | 1.10±0.10 (n=66) | 0.297 |
| Week 52 (change from baseline) | 0.01±0.11 (n=52) | 0.00±0.10 (n=53) | 0.124 |
| Week 104 (change from baseline) | 0.00±0.10 (n=46) | 0.00±0.10 (n=47) | 0.148 |
| DTR-QOL7 Q2 score | 4.8±1.8 (n=75) | 4.6±1.7 (n=77) | 0.366 |
| Week 26 (change from baseline) | 1.2±1.8 (n=58)§ | 0.3±1.9 (n=57) | 0.013 |
| Week 52 (change from baseline) | 0.5±2.1 (n=55) | 0.5±1.9 (n=61)* | 0.963 |
| Week 104 (change from baseline) | 0.4±2.1 (n=61) | 0.4±1.8 (n=55) | 0.977 |
| DTR-QOL7 total score | 59.9±19.5 (n=75) | 58.0±18.4 (n=77) | 0.558 |
| Week 26 (change from baseline) | 15.6±18.4 (n=58)§ | 1.2±17.1 (n=57) | < 0.001 |
| Week 52 (change from baseline) | 13.7±18.6 (n=55)§ | 3.9±15.2 (n=61) | 0.003 |
| Week 104 (change from baseline) | 12.1±17.1 (n=61)§ | 4.0±16.0 (n=58) | 0.009 |

Data are presented as means ± standard deviations. Differences in parameters between groups at baseline were analyzed using Student’s t-test. Differences in parameters from baseline to week 26, 52, 78, and 104 within each group were analyzed by one-sample t-test. Differences in parameters from baseline to week 26, 52, 78, and 104 between groups were analyzed using Student’s t-test. *P<0.05, ^#^P<0.01, §P<0.001.

HbA1c, glycated hemoglobin; AST, aspartate aminotransferase; ALT, alanine transaminase; γ-GTP, gamma glutamyl transferase; LDL-C, low-density lipoprotein-cholesterol; HDL-C, high-density lipoprotein-cholesterol; eGFR, estimated glomerular filtration rate; NT-proBNP, N-terminal pro-B-type natriuretic peptide; CCA, common carotid artery; IMT, intima-media thickness; BIF, bifurcation; ICA, internal carotid artery; baPWV, brachial-ankle pulse wave velocity; ABI, ankle brachial index; DTR-QOL, diabetes therapy-related quality of life.
